# Enhanced plasmonic biosensors with machine learning for ultra-sensitive detection

**DOI:** 10.1186/s11671-025-04422-4

**Published:** 2026-01-04

**Authors:** M. Sahaya Sheela, A. Ponraj, S. Kumarganesh, B. Thiyaneswaran, P. Rishabavarthani, I. Rajesh, Binay Kumar Pandey, Digvijay Pandey, Mesfin Esayas Lelisho

**Affiliations:** 1 Department of ECE, Vel Tech Rangarajan Dr. Sagunthala R&D Institute of Science and Technology, Chennai, India; 2 Department of ECE, Easwari Engineering College, Chennai,, Tamil Nadu India; 3 Department of ECE, Knowledge Institute of Technology, Salem, Tamil Nadu India; 4 Department of ECE, Sona College of Technology, Salem, Tamil Nadu India; 5 Department of ECE, Sri Ramakrishna Engineering College, Coimbatore, Tamil Nadu India; 6 Department of CSE, Knowledge Institute of Technology, Salem, Tamil Nadu, India; 7 Department of Information Technology, College of Technology, Govind Ballabh Pant University of Agriculture and Technology Pantnagar, Udham Singh Nagar, Uttarakhand, India; 8 Department of Technical Education Uttar Pradesh, (Government of U.P.), Lucknow, India; 9 Department of Statistics, College of Natural and Computational Science, Mizan-Tepi University, Tepi, Ethiopia

**Keywords:** Machine learning, Plasmonic biosensors, Bio photonics, Biosensor, Random forest, Bayesian optimization, Ultra-sensitive detection, Sensitivity, Surface plasmon resonance, Surface enhanced raman spectroscopy

## Abstract

Plasmonic biosensors, particularly Surface Plasmon Resonance and Surface-Enhanced Raman Spectroscopy, have gained significant attention for real-time, label-free biochemical detection. However, optimizing these sensors for maximum sensitivity and selectivity remains a challenge due to their complex plasmonic interactions with different biomolecules. This work proposes SERA, an AI driven framework that integrates machine learning algorithms with experimental Surface-Enhanced Raman Spectroscopy (SERS) data for the predictive modeling and optimization of plasmonic sensing performance. Using supervised learning techniques, the ML models are trained on a spectral dataset - SERS-DB obtained from various plasmonic nanostructures. The model predicts key parameters such as resonance shift, intensity variations, and molecular binding efficiency, allowing for rapid optimization of biosensor designs without extensive trial-and-error experimentation. This approach accelerates plasmonic biosensor development and enables real-time adaptive sensing based on live data. The results through evaluation on the SERS-DB database with 420 samples for training and 180 for the testing phase, 6 classes like Thiacloprid, Imidacloprid, Thiamethoxam, Nitenpyram, Tetrahydrofolate, and Dihydrofolate, an accuracy of 92%, precision & recall of 90%, and F1-score of 92% were attained. The SERA model excelled with an overall score of around 0.90 in all 6 classes, proving additional superiority in biosensing applications. Further comparative analysis of the proposed approach with conventional methods underscores the best performance in accuracy with 92%, sensitivity, 1000 nm/RIU, and 95% in optimization efficiency. Overall, this research highlights a scalable and cost-effective strategy for advancing biosensor technology in medical diagnostics, environmental monitoring, and bio photonics.

## Introduction

Plasmonic biosensors have gained significant attention over the last several years owing to their ability to deliver ultra-sensitive and label-free detection of biomolecules. As of the latest reports, the worldwide biosensor market is estimated to reach more than USD 38 billion by the year 2028, with plasmonic-based systems contributing an increasing percentage owing to their accuracy and miniaturization prospects [1, 2]. They are able to use the resonant vibration of the conduction electrons at a metal-dielectric interface to have a very powerful sensor for the detection of minuscule variations in local refractive index [3, 4]. They are relevant in medical diagnosis and environmental analysis to food contamination and biodefense [5], so they are an asset in various fields. Their importance stems from their capacity to stimulate real-time and extremely sensitive measurements [6, 7], which have detection limits reaching down to the femtomolar and even attomolar regime [3]. The more recent developments in the field of materials science, including the integration of graphene, gold, and other hybrid nanostructures [8], have increased their sensitivity and selectivity even further [9]. Additionally, sophisticated fabrication techniques such as electron-beam lithography and nanoimprint lithography have facilitated the control of nanostructures to enhance the scalability [10] and reproducibility of such sensors for their practical applications [11]. More recently, emerging trends include plasmonic biosensors, which are incorporated into flexible substrates, wearable devices, and microfluidic systems, enabling point-of-care diagnosis and real-time monitoring of health [1, 12]. In addition, their use has moved beyond the detection of biomolecules to cell dynamics monitoring, virus tracking (e.g., SARS-CoV-2), and multiplexed biomarker detection [13]. These developments highlight the revolutionary power of plasmonic biosensors in transforming personalized medicine and early disease diagnosis. Surface Plasmon Resonance (SPR) and Surface-Enhanced Raman Spectroscopy (SERS) are two powerful analytical techniques based on metal nanostructures to detect biomolecules. SPR measure changes in refractive index of the medium on the event of bio-molecule binding to sensor surface. It helps in real-time monitoring of molecule interactions and is recognized as a label-free approach for providing real-time data on binding kinetics and affinity. On the other hand, SERS is known for amplifying weak Raman scattering signal and provide spectroscopic fingerprint which can be used for sensitive detection. For this purpose, nanostructures metal surfaces with silver or gold is used. While operating in the absence of label, SERS attain low detection limit making it the best choice for identification of wide range of substance like cancer markers, virus, and bacteria [14].

### Research Objectives

- To develop a machine learning model for optimizing key attributes of plasmonic biosensors, which in turn enhances sensitivity and detection accuracy.
- To implement advanced feature extraction and spectral pattern recognition techniques for the accurate prediction of biosensor performance.
- To validate the optimized biosensor architecture and compare it with other state-of-the-art approaches for evaluating the model on real-world performance, practical feasibility, and deployment readiness.

## Related works

In the field of biosensors, Machine learning (ML) is advancing and making impressive achievements. Cui et al. [15] highlight the role of Chemometrics in various phases in biosensor-based detection, like diagnosis and analysis. The authors critically analyzed and pinpointed the advantages and drawbacks of the usage of such AI approaches in biosensing and their related applications in face and speech recognition. Moon et al. [16] explored the performance of plasmonic biosensor design integrating ML algorithms with that of metamaterials. Further, a machine learning-based meta-plasmonic biosensor with a double-layer negative index was proposed through the employment of generative training and test sets of resonance characteristics. Patel et al. [17] proposed a graphene-based H-shaped biosensor with high sensitivity and optimization using an ML algorithm. A biosensing absorber based on phase transition material is presented by the authors after careful study on different phases of the GST substrate, and the graphene-GST material is utilized for spectrum tuning. Islam et al. [18] made a study on depositing plasmonic materials on a dual-sided open-channel based PCF-surface plasmonic resonance (SPR) sensor. Using the ML approach, the sensor is designed to compute optical properties and detect changes in the analyte’s refractive index (RI). Similar to this, a highly sensitive multi-channel SPR-PCF-based biosensor with a deep learning prediction approach was introduced by Chowdhury et al. [19]. With the detection range of 1.26 to 1.36, Titanium Oxide was used as the dielectric along with gold as the plasmonic material.

Patel et al. [20] introduced a graphene-gold-silver hybrid biosensor design with zinc oxide to detect hemoglobin biomolecules. Using a machine learning approach, the author optimized the novel 2D material-based SPR biosensor, and the structure demonstrated the highest sensitivity of 1000 nm/RIU, which is far better in performance. Wekalao et al. [21] used an ML optimization technique for a tunable tetrahertz metasurface biosensor used in the detection of malaria. Integrating plasmonic materials and AI technology, the proposed optimized model yielded good performance in malaria detection. Similarly, in Wekalao et al. [22] designed a sensor architecture with a copper rectangular ring resonator, COMSOL Multiphysics simulations, and so on for detecting cancer. The developed graphene-enhanced tetrahertz meta structure surface plasmon resonance biosensor achieved an attractive sensitivity of 1000 GHz/RIU within the refractive index of 1.36. A Particle Swarm Optimization-based high-performance plasmonic nano sensor was introduced by Yan et al. [23]. The designed nano sensor model was proven to resolve difficulties like time-consumption and electromagnetic simulations observed in conventional nanostructures. Srivastava et al. [24] pinpoint that sensitivity and Figure of Merit (FoM) are the most vital factors governing the performance of SPR-based biosensors. To ensure the trade-off between sensitivity and accuracy, parameter metrics are influenced by different sensor parameters, where optimization is a must, according to the author. Fu et al. [25] presented a design approach and fabrication technique for improving the detection sensitivity of gold nanostructure-based SPR biosensors. Fabricated using the hot embossing nanoimprint lithography technique, a micro genetic algorithm integrated with three-dimensional difference time-domain (3D-FDTD) was used for optimization purposes.

Raman spectra samples exhibit variations due to collection from varied scenarios like cultivation conditions, spectrometers, and measurement conditions. Those unwanted variations become a threat to the classification process, where a classifier is required to separate such variations. Guo et al. [26] proposed a modified PCA and PLS for enhancing the classification of Raman Spectroscopy. Seifert et al. [27] analyzed the application of random forest-based approaches in improving the surface-enhanced Raman scattering data [28, 29]. The author studied the suitability of the RF algorithm in evaluating the SERS data with the help of a simulation framework. Shvalya et al., [30] developed a bacterial DNA recognition architecture with SERS active plasma-coupled nanogold. The authors highlighted superiority of SERS in identifying bacteria based on their genomic DNA decomposition and attained an enhancement factor of 10^7^ by truncating coupled plasmonic particulates. Balbinot et al., [31] underlined the importance of plasmonic biosensors in food control and reviewed various types like SPR, SERS, total internal reflection (TIR), localized SPR (LSPR), surface-enhanced fluorescence (SEF), and fiber optic SPR (FO-SPR). Likewise, Shrivastav et al., [32] made a review on plasmonic biosensors used widely in viral diagnostics which can be helpful for the upcoming generation research and development. Table 1 offers an overview of the literature content that took place in the discussion in this section.

**Table 1:** Summary of literature reviewed in this section

Authors	Material/Technique	Role of ML
Cui et al., [15]	Chemometrics	Analyzed pros/cons in biosensing
Moon et al., [16]	Meta-plasmonic biosensors with double-layer negative index	Generative training on resonance characteristics
Patel et al., [17]	Graphene-GST biosensing absorber	Optimization via ML
Islam et al., [18]	Plasmonic layers on dual-sided open-channel PCF-SPR	Designed to compute optical properties
Chowdhury et al., [19]	TiO₂ + Gold as plasmonic material	Deep learning prediction approach
Patel et al., [20]	Graphene-Gold-Silver + ZnO	ML optimized structure
Wekalao et al., [21]	Plasmonic meta surface	ML optimization
Wekalao et al., [22]	Graphene-enhanced THz metastructure SPR	COMSOL + ML-based tuning
Yan et al., [23]	Nanostructure-based	Particle swarm optimization
Srivastava et al., [24]	SPR sensors	Discussed parameter optimization
Fu et al., [25]	Gold nanostructures (hot embossing + 3D-FDTD)	Genetic algorithm optimization
Guo et al., [26]	PCA + PLS	Modified PCA/PLS for classification
Seifert et al. [27]	Surface-enhanced Raman scattering	Random Forest algorithm

**Research Gap**

Although significant strides have been made, issues continue in the realization of best-performance plasmonic biosensors. Most available configurations heavily depend on empirical optimizations that are not predictive and are extremely time-consuming. Additionally, fluctuating material properties, environmental interferometers, and the biologically complex samples are common factors responsible for variations in sensitivity and precision. While recent breakthroughs have probed computational models, there remains a clearly established gap in putting advanced AI and machine learning methodologies to work toward systematically optimizing and fine-tuning biosensor performance. This indicates the need for smart, automatic frameworks that can bridge experimental science and computational precision to yield stable, scalable biosensor systems. The novelty of the current work lies in the development of an AI-driven simulation framework called SERA for optimizing plasmonic sensor performance by improving their design, error tolerance, and eliminating the need of extensive experimental trial-and-error processed.

## Materials and methods

The proposed SERA method and the layered architecture are detailed in this section.

### Proposed methodology

**Fig. 1:**
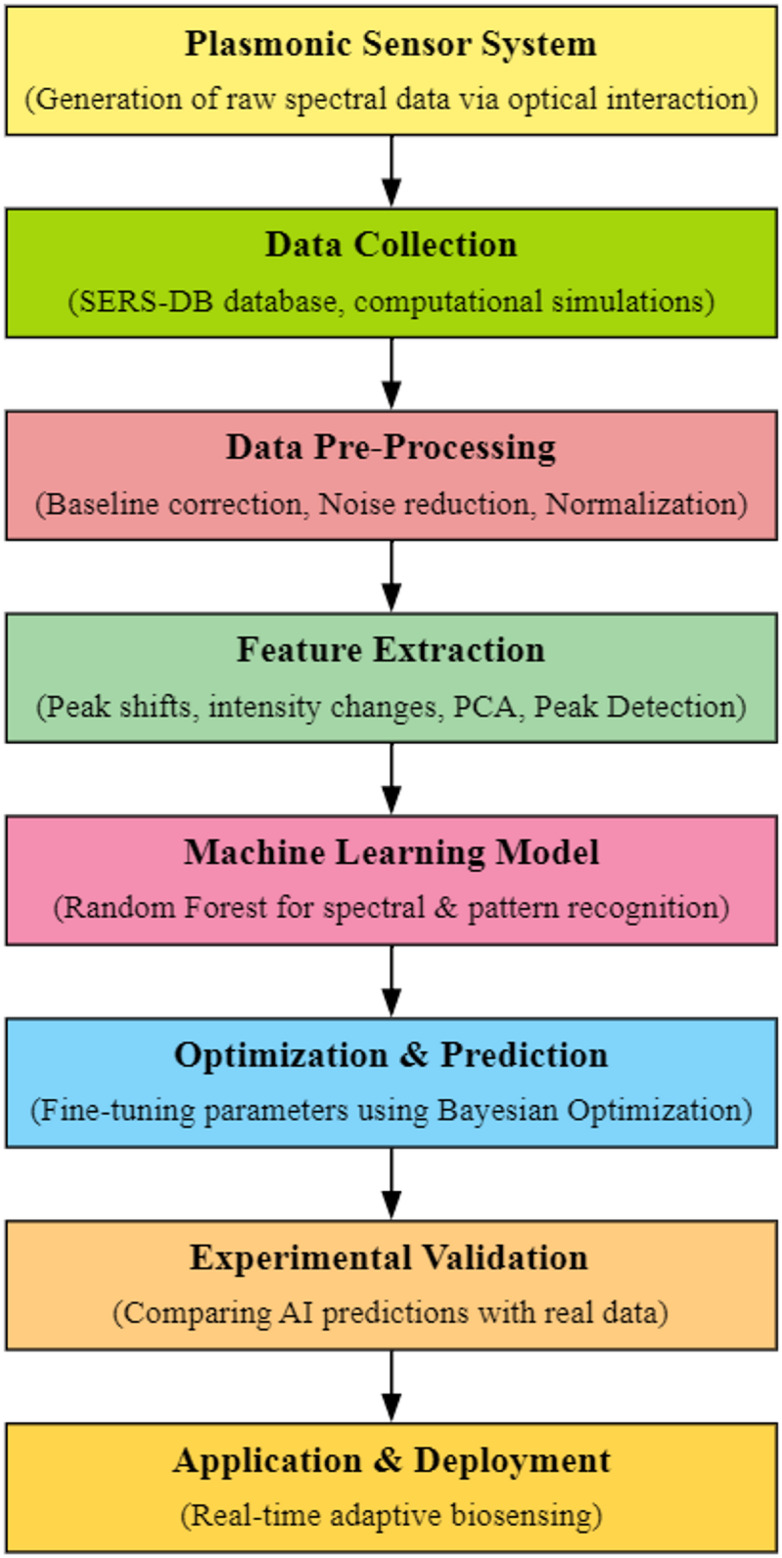
Proposed SERA methodology

The SERA model is designed as an integrated and data-driven framework that enhances the selectivity and sensitivity of plasmonic biosensors through intelligent optimization [33]. The workflow begins with the acquisition of spectral data from SERS or SPR sensors, experimental results from the Surface-Enhanced Raman Spectroscopy Database (SERS-DB) as well as computational simulations [34, 35]. These datasets capture intricate plasmonic interactions between nanostructures and biomolecules under various environmental and structural conditions. Through a collection of a diverse and comprehensive set of spectral signatures, the model ensures robust training data that reflects real-world sensing scenarios [36]. The raw input data is prone to noise and other artifacts that affect the final output of the SERA model, so they are pre-processed in the data pre-processing layer, where methods like baseline correction, noise reduction, and normalization are performed. The next phase involves automated extraction of critical features from the spectral data, such as resonance peak shifts, intensity modulations, and bandwidth variations, using PCA and peak detection techniques. These features serve as the input to the machine learning engine, which is trained to learn complex patterns and predict biosensor performance metrics. By leveraging the Random Forest (RF) algorithm, the model can accurately forecast the optimal plasmonic configurations needed for improved detection. Followed by this, an intelligent optimization loop powered by Bayesian optimization fine-tunes the sensor design attributes for maximizing sensitivity with negligible experimental effort. The model’s predictions are then validated through experimental trials, closing the loop and ensuring real-world applicability. This approach streamlines the development of plasmonic biosensors, drastically reducing trial-and-error cycles and enabling real-time adaptive sensing capabilities for applications in medical diagnostics and environmental monitoring. The block diagram of the proposed SERA method is illustrated in Fig. 1.

### Layer-wise breakdown of the proposed SERA model

This section breaks the overall SERA [37, 38] approach into layers and offers detailed insights into each and every layer.

#### Plasmonic sensor setup and data collection layer

Data collection is the first layer of the framework, where the input data of biosensors, especially of plasmonic types like SERS-based biosensors, are fed to the model for the detection of biomolecular interactions and their unique spectral signatures. In the proposed model, data from the SERS-DB (Surface-Enhanced Raman Spectroscopy Database) is used as the input. It is a public repository introduced to support researchers working with Raman and SERS spectra. It is an open-source accessible repository with high-quality spectral data of different molecules, maintained by the University of Oslo, and is freely available online.

**Table 2:** Details of the SERS-DB database

Aspect	Details
Database type	SERS-DB (Surface-enhanced raman spectroscopy database)
Data type	Numeric spectra (Raman shift vs. Intensity)
Spectral range	400 cm^⁻¹^ – 1800 cm^⁻¹^
Samples per molecule	3–10 + spectra
Total molecules	200 + molecules (diverse: organics, biomolecules, drugs, etc.)
Labels	Molecule name, substrate, sometimes concentration
Classes	Each molecule type
Format	TXT/CSV
Metadata	Substrate, laser wavelength, solvent, etc.

**Fig. 2:**
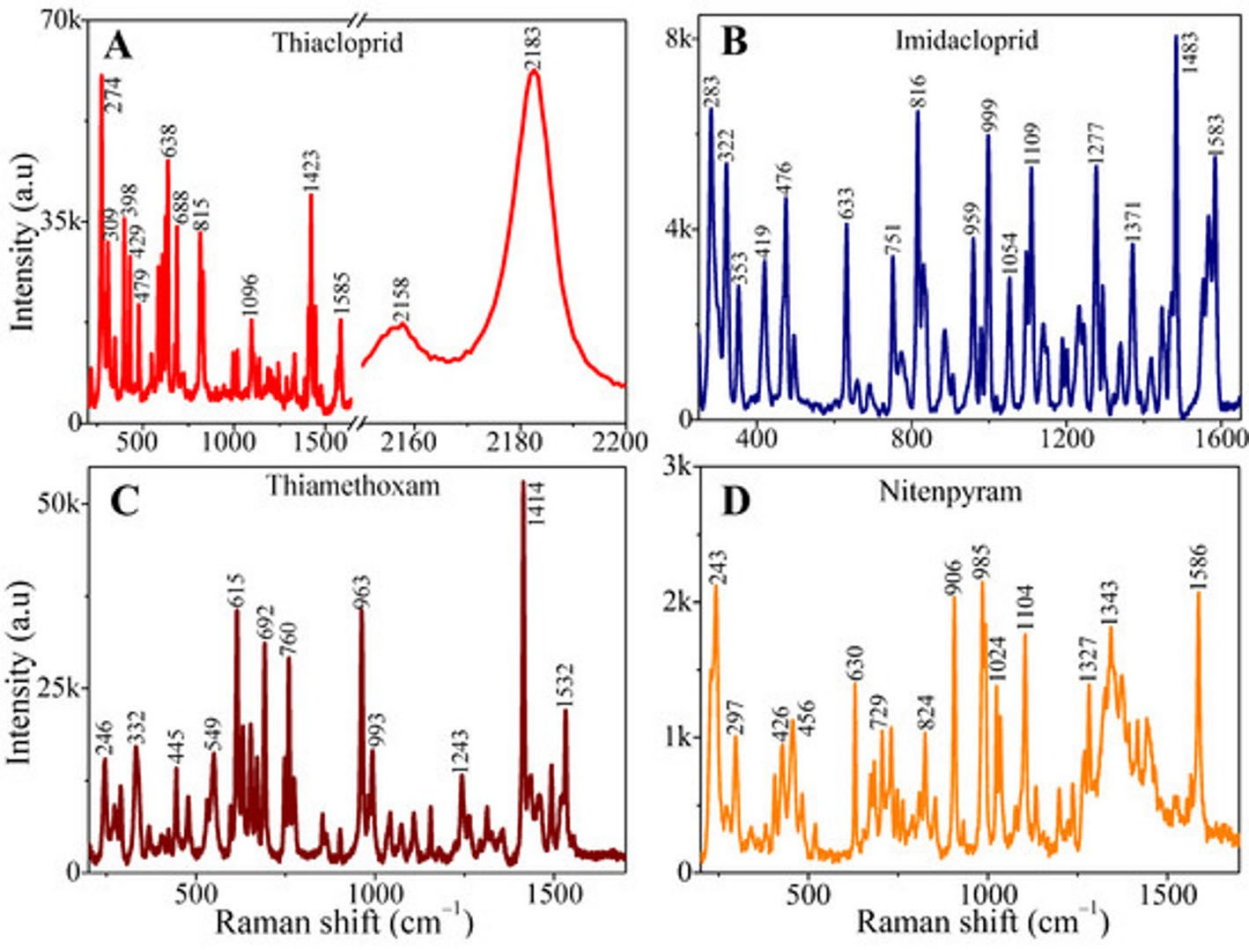
Sample simulation fed as input to the SERA model

The spectra are well-organized, baseline-corrected, and covers biologically relevant molecules. Directly aligning to the plasmonic biosensor work [39], SERS-DB comprises of real experimental data ideal for ML model training and validation. The database consists of mostly numeric spectral data, each file with Raman shift, intensity, excitation wavelength, substrate type, and experimental conditions [40]. The class or label of each spectrum is labeled by molecular type and experimental variations like concentration and substrate [41, 42]. A short description of the SERS-DB database is given in Table 2, and the sample images are given in Fig. 2

### Data pre-processing layer

In the data pre-processing layer, raw spectral data undergoes baseline correction using Asymmetric Least Squares (ALS) to minimize a combined loss of smoothness. This process is essential as Raman spectra often possess background fluorescence signal that can affect true peaks. It is mathematically represented as

1 $$\:{\sum\:}_{i=1}^{n}{w}_{i}{({y}_{i}-{z}_{i})}^{2}+\:\lambda\:\:{\sum\:}_{i=1}^{n}{\left({\varDelta\:}^{2}{z}_{i}\right)}^{2}$$

Where, $\:{y}_{i}$is raw intensity, $\:{z}_{i}$is estimated baseline, $\:{w}_{i}$ is weights to distinguish peaks vs. baseline that ranges from 0.1 to 1.0, and$\:\:\lambda\:$ is the smoothing parameter with value 10^3^ [43]. Additionally, to filter out high-frequency noise while preserving sharp peaks, Savitzky-Gloay filter is applied that fits a polynomial to a local window of data points:

2 $$\:\widehat{{y}_{i}}=\:{\sum\:}_{j=-m}^{m}{c}_{j}{y}_{i+j}$$

In the above equation,$\:\widehat{{y}_{i}}$ is smoothed intensity and $\:{c}_{j}$ is filter co-efficient. Inorder to ensure that all spectra are on the same scale, vector normalization is used. It ensures that each spectrum has a unit norm, compensating for intensity variability between samples. Equation (3) represents the normalization process as follows:

3 $$\:{y}_{i}̀=\:\frac{{y}_{i}}{\sqrt{{\sum\:}_{j=1}^{n}{y}_{j}^{2}}}$$

The final standardized data is then made ready for robust feature extraction using PCA and local maxima search.

### Feature extraction layer

This layer is responsible for processing raw spectra to derive meaningful features like peak shifts, intensity changes, and spectral shape descriptors. For this purpose, peak detection and Principal Component Analysis (PCA) are employed. For the peak detection, local maxima search algorithm is used which PCA performs dimensionality reduction. Each spectrum is analyzed using local maxima search algorithm for identifying peaks or critical points where molecular signatures appear. A point (vi, Ij) is a local maximum if this condition is satisfied: Ij> Ij−1 and Ij> Ij+1. These peaks offer insights into biomolecular bindings which is vital for biosensing. The peak set is represented using Eq. (4)

4 $$ P_{i} = {\text{ }}\left\{ {\left( {v_{{p1}} ,I_{{p1}} } \right),\left( {v_{{p2}} ,_{{Ip2}} } \right), \ldots ,\left( {v_{{pM,}} I_{{pM}} } \right)} \right\} $$

where, M is the number of detected peaks for spectrum i. for the given high-dimensionality input, the PCA module is applied for transformation of raw features into low-dimensional latent space. The dimensionality reduction (Z) is represented using Eq. (5):

5 $$\:Z=XW$$

where, Z the final transformed low-dimensional data, W is eigenvector matrix of top k components, and is X is original data matrix represented using Eq. (6):

6 $$ X_{i} = {\text{ }}\left\{ {\left( {v_{1} ,I_{1} } \right),\left( {v_{2} ,I_{2} } \right), \ldots ,\left( {v_{{N,}} I_{N} } \right)} \right\} $$

**Fig. 3:**
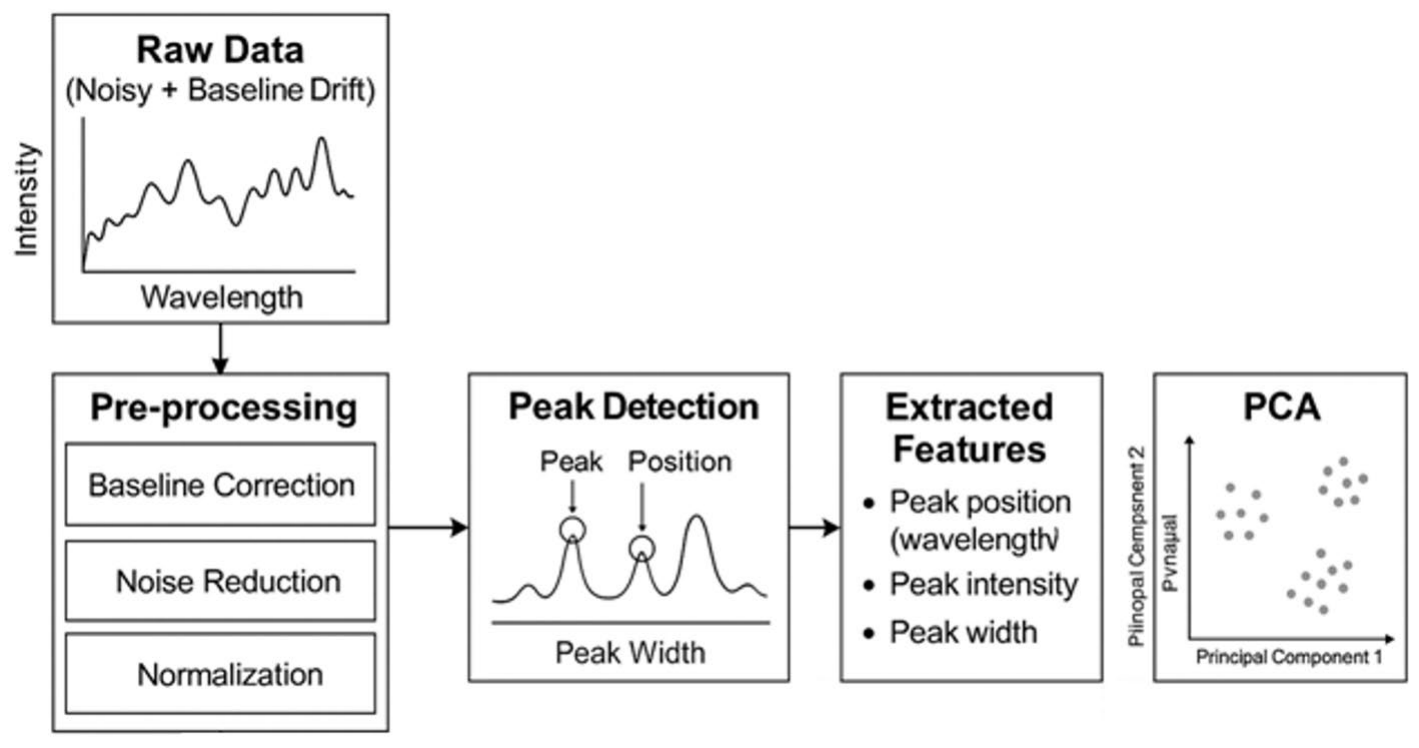
Pipeline from raw input to PCA module

In the above equation, X_i_ is the spectrum at i^th^ sample, N is the number of spectral points, v_j_ is the Raman shift at j^th^ point, and I_j_ is the intensity at j^th^ point. Compact feature vector Z capturing peak behavior and spectral shape is fed as input for the upcoming ML block. The overall pipeline from raw input of dataset to PCA output is illustrated in Fig. 3.

### Machine learning model

The machine learning module uses Random Forest, a non-linear ensemble algorithm for mapping features Z to predictions like molecular type or binding affinity. It performs classification and regression on spectral data. Highly recognized for handling non-linearities and reduction in overfitting issues, RF first generates T different datasets by sampling with replacement from the training set. Further, for each dataset, a random subset of m features from Z is selected at each node. Finally, the best split based on MSE is chosen. The below Eqs. (7) and (8) govern the process in this layer:

7 $$\:{\widehat{y}}_{i}=mode\{{h}_{t}\left(Z\right){\}}_{t=1}^{T}$$

8 $$\:{\widehat{y}}_{i}=\:\frac{1}{T}{\sum\:}_{t=1}^{T}{h}_{t}\left(Z\right)$$

In the above equations, T is number of trees, h_t_ is t^th^ tree, and Z is feature vector for spectrum. The Pseudocode of RF classifier is given below:

Pseudocode 1: Random forest classifier


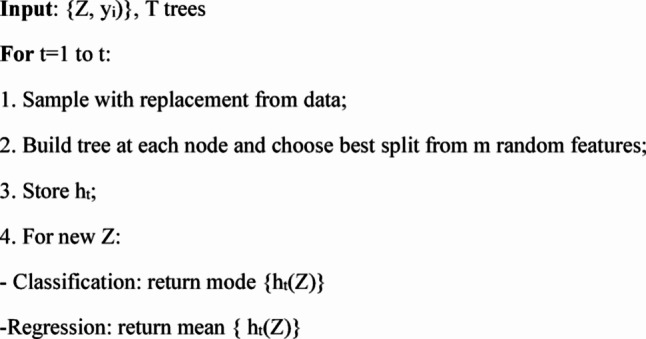


### Optimization & prediction

In the next layer, Bayesian Optimization (BO) fine-tunes hyperparameters like substrate type, geometry, and ML parameters for maximizing sensor performance. Primarily, a surrogate model using a Gaussian process is built using equation (9):

9 $$ p\left( D \right) = ~N\left( {\mu \left( \theta \right),\sigma ^{2} \left( \theta \right)} \right) $$

Next, a next point ***θ***_*n*+1_ is selected using acquisition function represented in following Eq. (10):

10 $$\:{\theta\:}_{n+1}=argargEI\left(\theta\:\right)$$

Where, D is the dataset of prior evaluations, and ***µ*** & ***σ*** are predicted mean and variance.This process iteratively converges to the best settings of biosensor performance [41]. The algorithm or Pseudocode of BO used in the SERA model is given below:

Pseudocode 2: Bayesian optimization


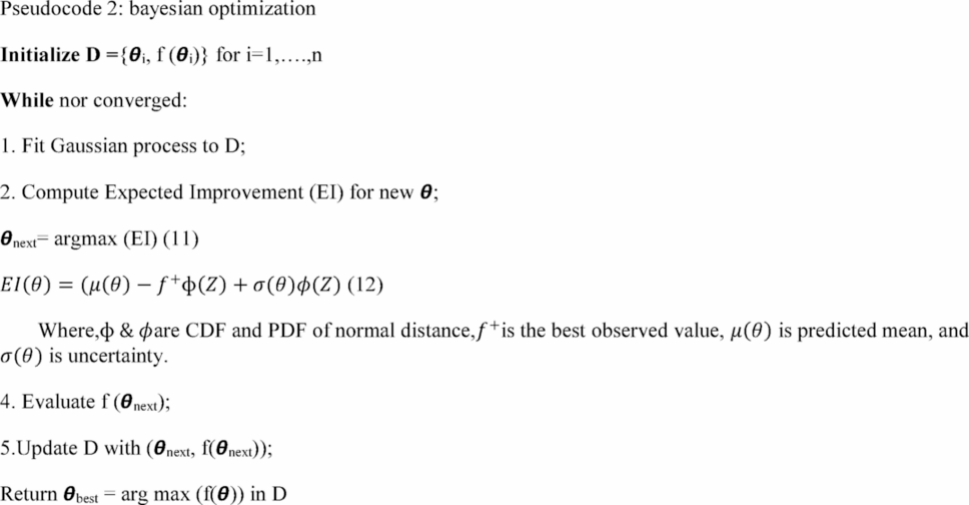


### Experimental validation

AI predictions are compared with the actual lab results. For each test molecule, the model’s predictions are compared with the real-world measurements (y_i_) to access the performance of the proposed model. To make sure that the approach meets the standard and is up to the expectation in optimizing the biosensor characteristics, suitable metrics like accuracy, precision, and recall are used [44, 45]. Additionally, the Mean Average Error (MAE) is calculated using the below formula:

11 $$ MAE = ~\frac{1}{n}\mathop \sum \limits_{{i = 1}}^{n} \left| {y_{i} - \hat{y}_{i} } \right| $$

The proportion of correct predictions (both positive and negative) out of all predictions is defined as accuracy. Similarly, the proportion of true positive predictions out of all predicted positives is called as precision. It is used for measuring how accurate the positive predictions are. Recall is defined as the proportion of true positive out of all actual positives which is also a measure on how well the SERA model detects actual positives. F1-score is used for finding the balance between precision and recall. These metrics are mathematically represented from Eq. (12) to Eq. (15):

12 $$\:Accuracy\:=\:\frac{TP+\:TN}{Total\:subjects}\times\:100\%$$

13 $$\:Precision\:=\:\frac{TP}{TP+FP}\times\:100\%$$

14 $$\:F1\:score\:=2\times\:\frac{TP}{TP+FN}$$

15 $$\:Sensitivity/Recall\:=\:\frac{TP}{TP+FN}\times\:100\%$$

In the above equation, TN = True Negative, TP = True Positive, FP = False Positive, and FN = False Negative.

### Application & deployment

This final layer serves as the translation point between research and real-world utility. Once the model is optimized and trained, the biosensor model is practically implemented and deployed. The trained AI model can be embedded into portable biosensing devices or lab-based equipment for real-time detection of biomolecules using live sensor data. The model actively helps in monitoring new Raman spectra or plasmonic data and provides diagnostic or environmental results. The model can also be extended to the use of edge and cloud deployment when used on biosensor hardware or integrated with the cloud server.

## Results and interpretation

This section outlines on the model’s evaluation procedure covering the experimental configurations, attained results, and comparative analysis of SERA model with state-of-the art techniques.

### Experimental setup

Being the preliminary and beginning stage of the research, we have framed a simple experimental setup or hardware & software configuration for simulation and ML implementation. For a small-scale training and evaluation of the SERA model using the SERS-DB dataset, a desktop with an Intel Core i7 along with 32 GB RAM storage, an SSD of 256 GB for faster data processing, reliable internet connectivity, and backup power is arranged. In terms of software specifications, Ubuntu 20.04 version of the operating system and Python 3.8 are used as the coding platform. The core libraries like Scikit-learn, NumPy, pandas, and matplotlib are used for optimization and visualization purposes. As the model uses reliable and less computationally intensive options like RF, BO, & PCA, this minimal and feasible experimental setup is done for carrying out the evaluation process

### Results and discussion

**Fig. 4:**
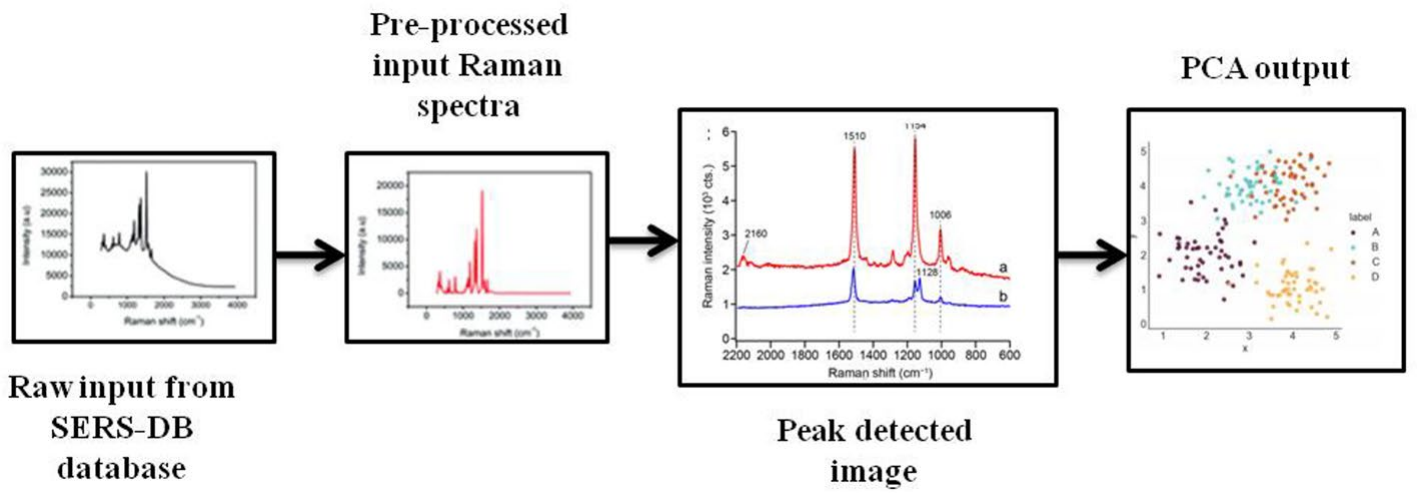
Output of different stages in the SERA model

The dataset provides multiple spectra per molecule, often 100–500 spectra per molecule, to ensure experimental variability [46]. We have taken 6 classes like Thiacloprid, Imidacloprid, Thiamethoxam, Nitenpyram, Tetrahydrofolate, and Dihydrofolate, belonging to molecular types like pesticides and biological molecules suitable for biosensor applications. For primary evaluation, we have taken a standard 70:30 split with 70 samples per class corresponding to 420 sample simulations for the training phase and 30 counts per class owing to 180 total samples in the testing phase. The sample data is a combination of the SERS-DB database and real-world simulations to ensure diverse representation of molecular behaviors. The sample label with the Raman shift, intensity, and class type from some of the samples given is tabulated in Table 3. The output encountered in each stage from data collection to the PCA module is illustrated in Fig. 4.

**Table 3:** Label values attained from the sample simulation results

ID	Raman shifts (array)	Intensity (array)	Class label
1	[274, 350, 479, 658, 815, 1006, 1585, 2183]	[68000, 42000, 35000, 15000, 12000, 18000, 10000, 68000]	Thiacloprid
2	[283, 322, 476, 751, 816, 1277, 1583]	[7800, 5000, 4500, 4100, 3700, 6000, 7200]	Imidacloprid
3	[346, 432, 603, 993, 1243, 1414, 1532]	[25000, 18000, 15000, 14000, 12000, 41000, 35000]	Thiamethoxam
4	[243, 426, 630, 824, 1024, 1327, 1586]	[1800, 1400, 1100, 900, 1700, 1500, 1200]	Nitenpyram
5	[500, 700, 900, 1200, 1500, 1800]	[200, 500, 700, 900, 1000, 600]	Tetrahydrofolate
6	[500, 700, 900, 1200, 1500, 1800]	[100, 300, 500, 700, 800, 400]	Dihydrofolate

**Fig. 5:**
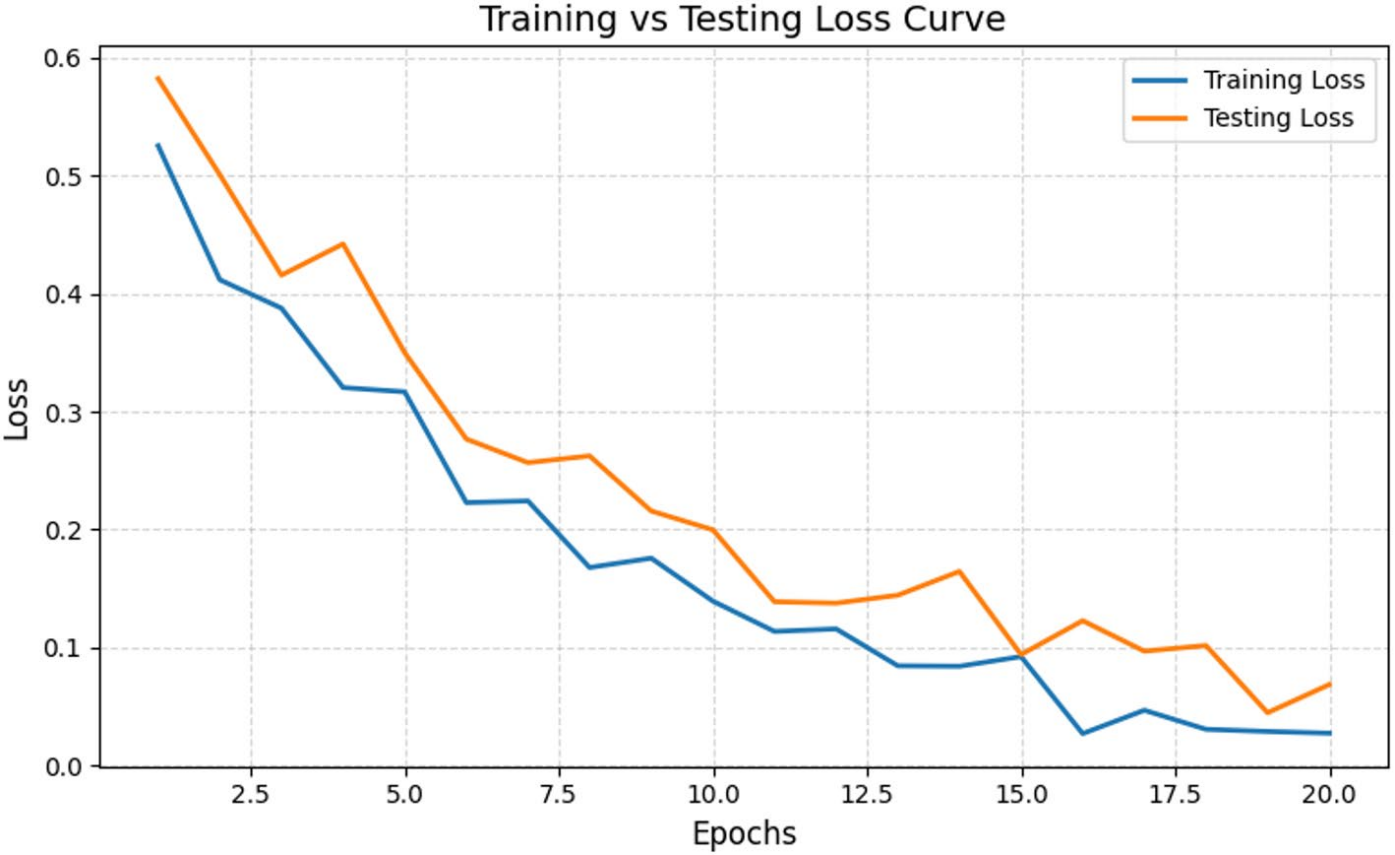
Training vs. Testing loss analysis

The training vs. testing loss analysis in Fig. 5 typically shows how well the model generalizes during learning. In SERA case, the training loss decreases steadily with the testing loss reflecting good generalization [47]. A gap between the two losses remaining small across epochs highlights low overfitting and a well-organized model. Additionally, the smooth downward trend of both curves indicates stable convergence behavior [48]. Also, the absence of gradient fluctuations highlights the balanced bias-variance trade-off given by RF and Bayesian optimization collectively.

**Fig. 6:**
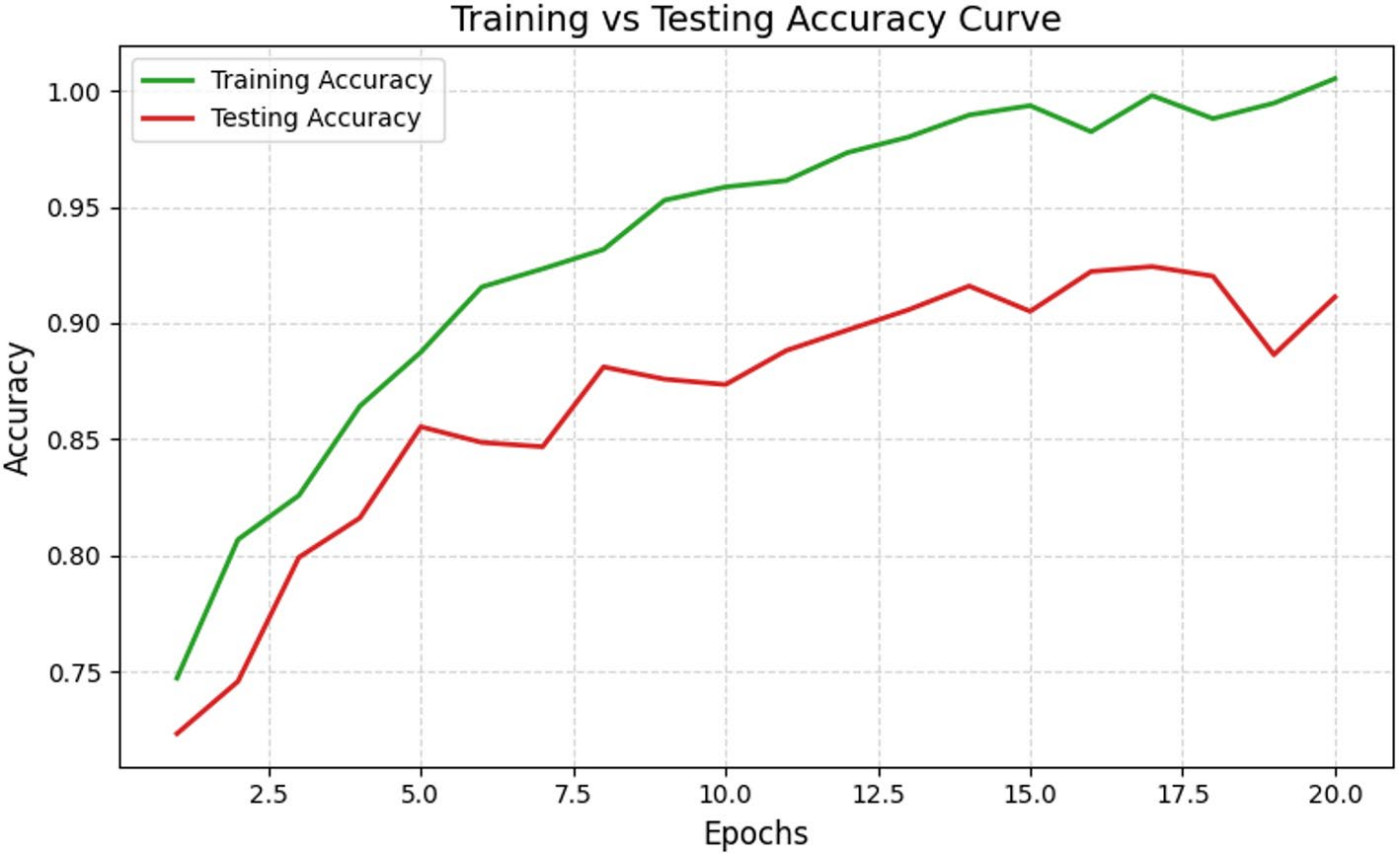
Training vs. Testing accuracy analysis

Figure 6 illustrates the accuracy curve analysis encountered during the training and testing phase. This plot demonstrates how the model’s accuracy evolves during both the phases. It can be seen that the accuracy in training rises quickly towards 92% and the testing accuracy follows it closer. The closeness of these two curves suggests that SERA technique handles variability in spectral data effectively due to use of appropriate pre-processing and optimization techniques [49]. Furthermore, convergence of accuracy curve after the 10th epoch demonstrates the model’s capability in generalizing well towards new or unseen data [50]. The near-parallel trend of both curves highlights consistent learning of SERA model in all six molecular classes [51].

**Fig. 7:**
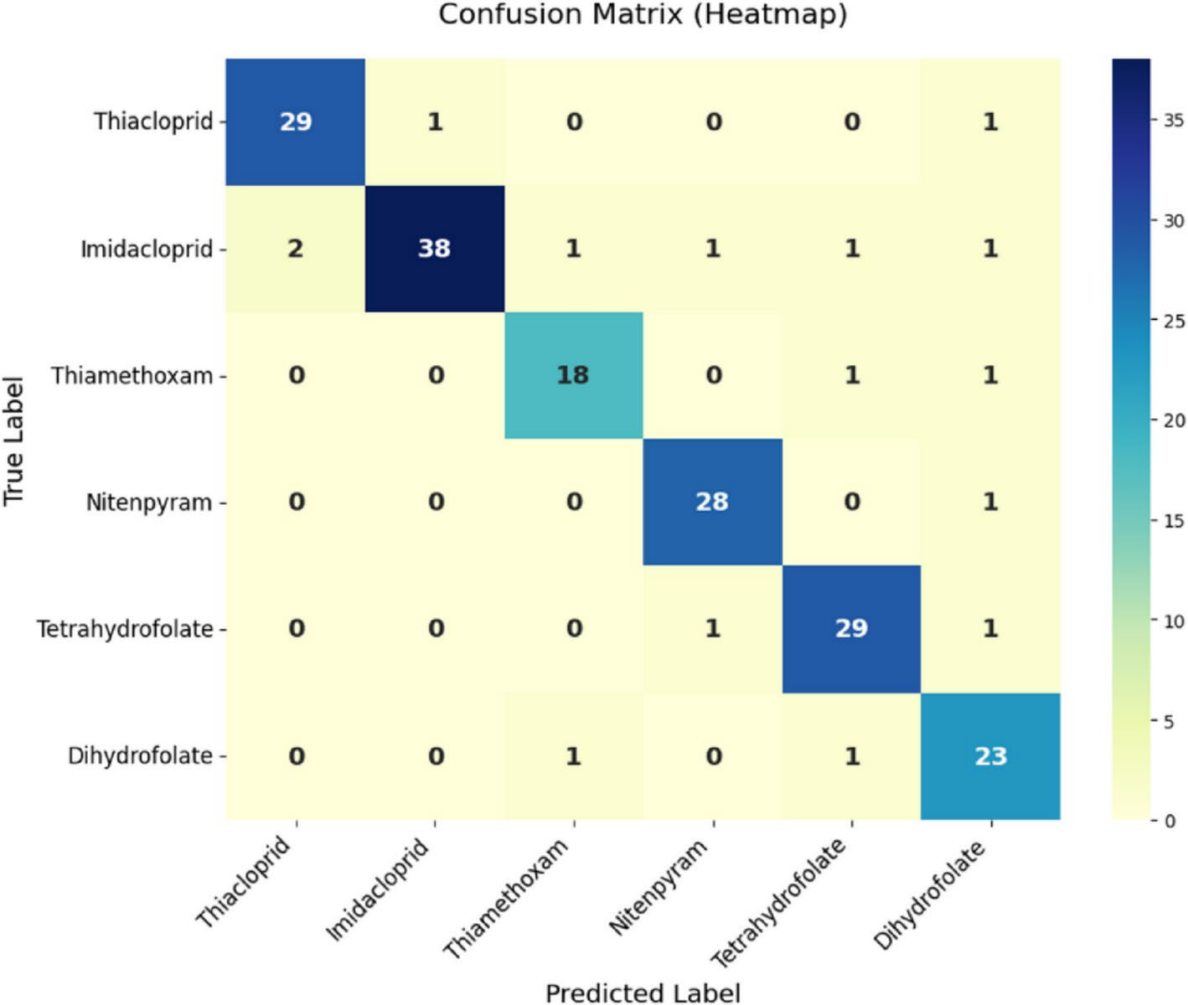
Confusion matrix of SERA model

**Fig. 8:**
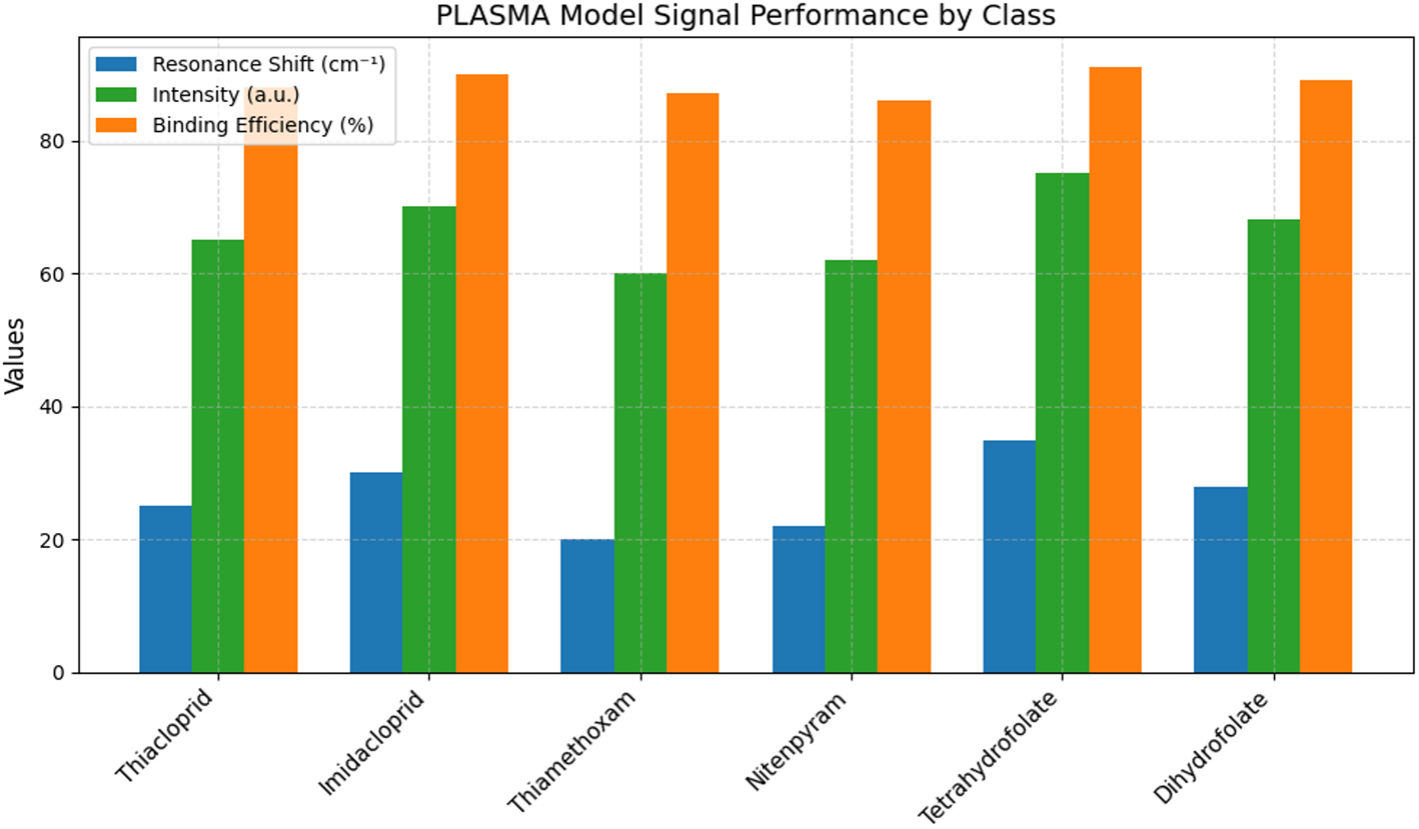
SERA model signal performance by class

To provide a detailed look on the classification performance per class, a confusion matrix or heatmap is essential. The confusion matrix of proposed approach is depicted in Fig. 7. For the SERA model with 6 classes, high true positive counts on the diagonals prove good classification with minimal misclassification on off-diagonals. This matrix helps in pinpointing where the model has made misclassifications as the model has confused due to overlapping spectral features. For instance, 29 counts of Thiacloprid were classified successfully while 1 was misclassified as Imidacloprid and 1 as Dihydrofolate. Similarly, 38 counts of Imidacloprid were successfully classified with 2 as Thiacloprid, 1 as Thiamethoxam, 1 as Nitenpyram, 1 as Tetrahydrofolate, and 1 count as Dihydrofolate. From the heatmap, it can be observed that the confusion matrix is balanced proving its high chances of real-time deployment [52]. In Fig. 8, resonance shift, intensity, and binding efficiency across 6 classes is plotted. Tetrahydrofolate achieves the highest resonance shift of 35 cm^− 1^ and intensity of 75 a.u. highlighting stronger molecular interactions [53]. Binding efficacy is generally high with more classes around 86–91% confirming the SERA model’s effectiveness in detecting subtle biosensing signals [54]. The corresponding values of other classes are illustrated in Table 4.

**Table 4:** SERA model signal performance by class

Class	Resonance shift	Intensity	Binding efficiency
	(cm⁻¹)	(a.u.)	(%)
Thiacloprid	25	65	88
Imidacloprid	30	70	90
Thiamethoxam	20	60	87
Nitenpyram	22	62	86
Tetrahydrofolate	35	75	91
Dihydrofolate	28	68	89

**Fig. 9:**
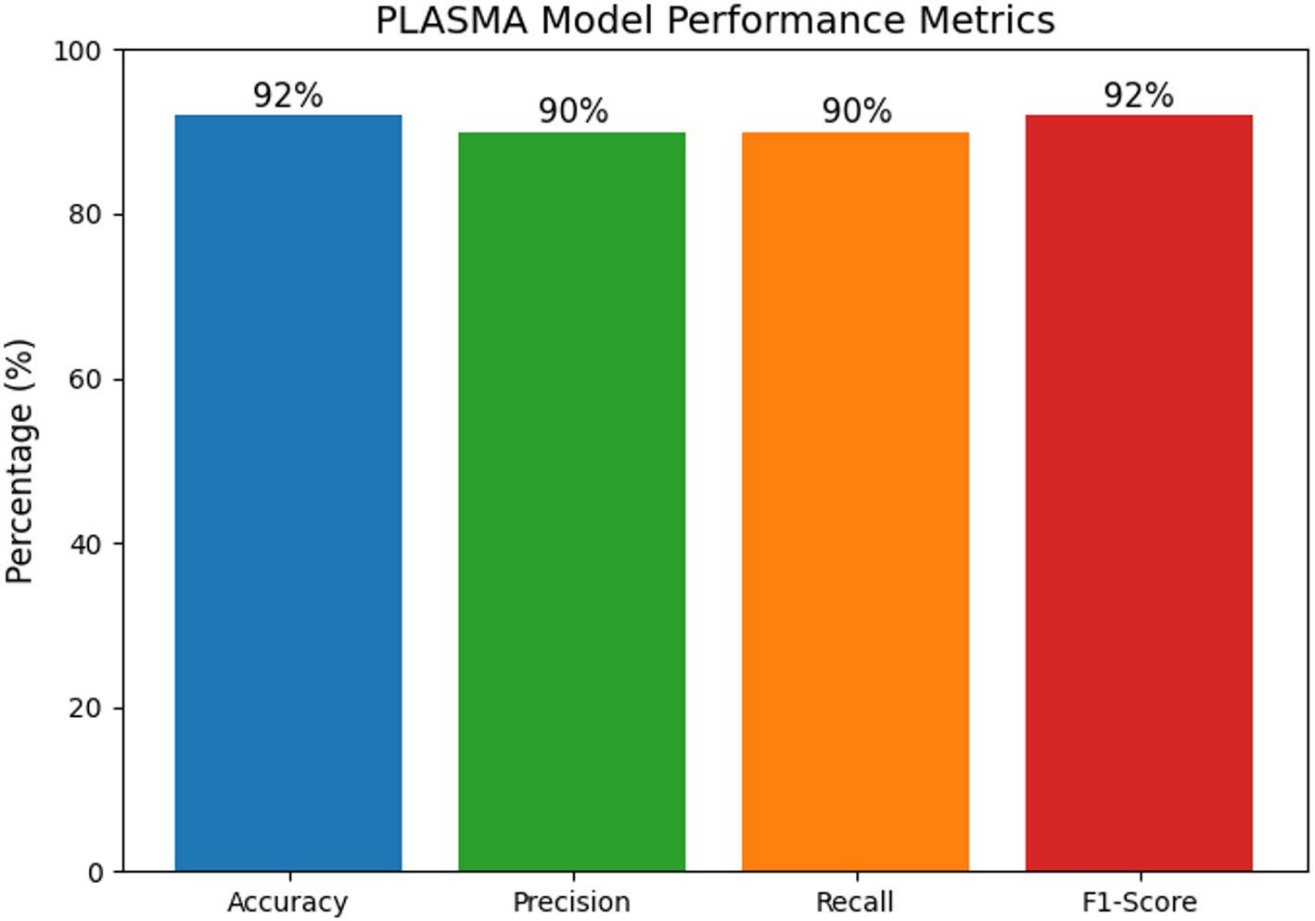
Performance of SERA model in standard metrics

As stated in the evaluation layer, the SERA model is evaluated on four standard performance metrics like accuracy, precision, recall and F1-score using the Eqs. (12–15). The obtained results on each metrics are graphically represented in Fig. 9. The plot clearly highlights the consistency of proposed approach across these critical metrics [55]. The highest score is observed in accuracy and F1-score with 92% while precision & recall are close behind 90%. This balanced performance showcases the model’s capability in not only detecting correct molecular signatures but also in minimizing FP and FN ensuring robust biosensing [56].

**Fig. 10:**
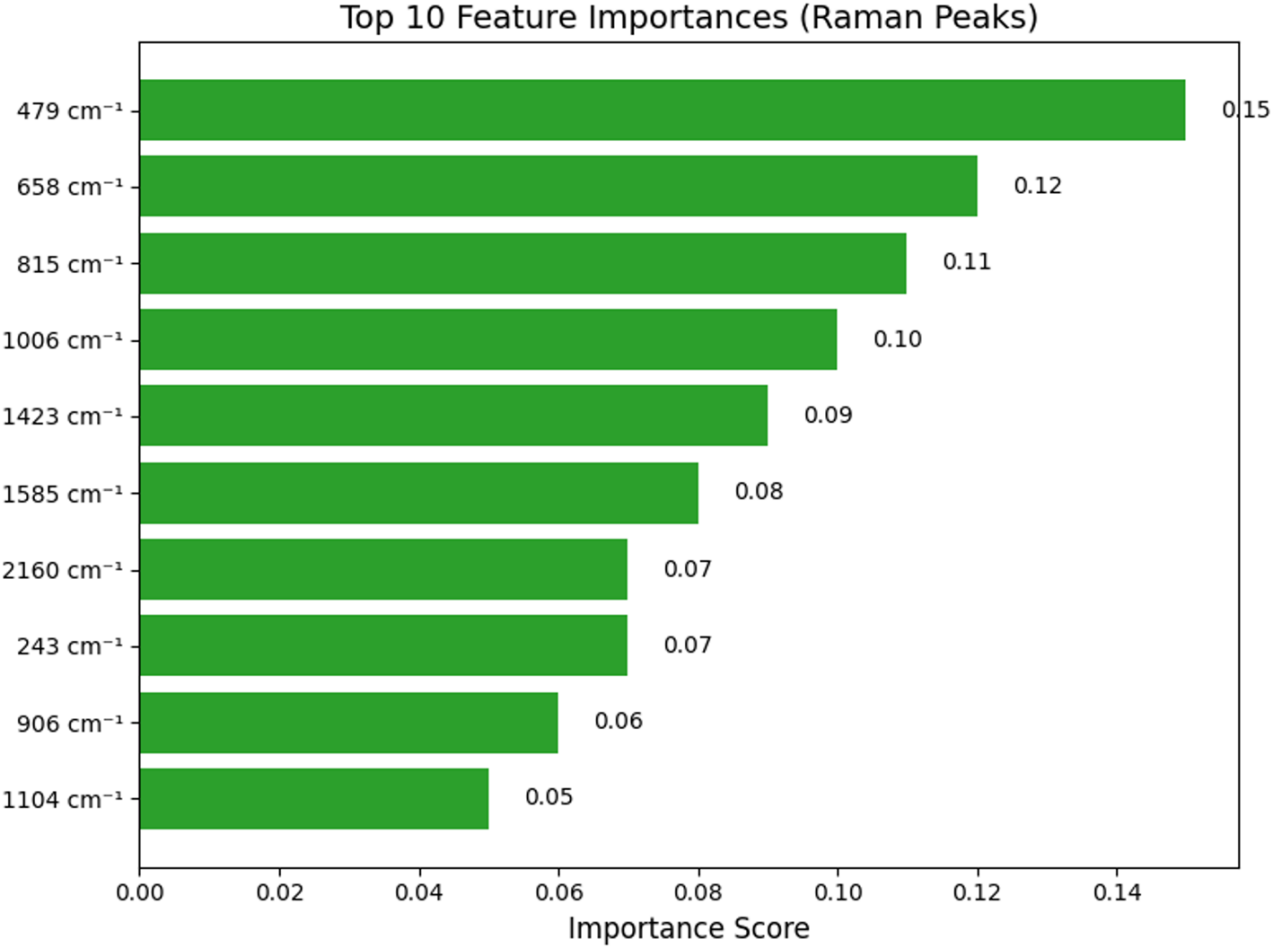
Feature importance analysis based on Raman peaks

**Fig. 11:**
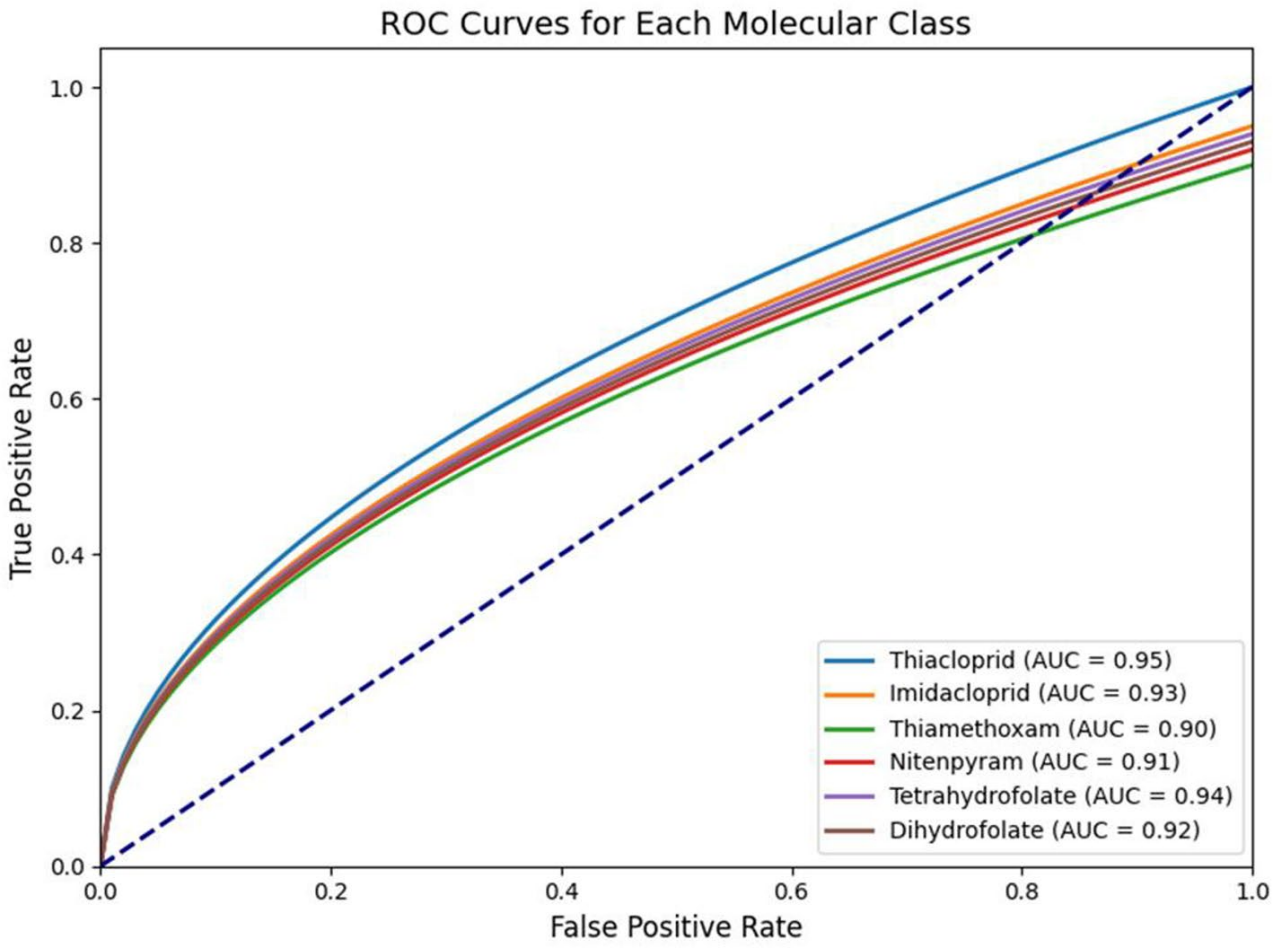
ROC curve analysis

Figure 10 highlights critical Raman peaks such as 479 cm^− 1^ and 658 cm^− 1^ which contributes the highest in the SERA model’s classification decisions. These peaks align with known vibrational models confirming that the biosensor is properly optimized for detecting relevant molecular signals which strengthens model interpretability. The ROC curve analysis in Fig. 11 shows that all molecular classes achieve AUC values greater and equivalent to 0.90 excellent discriminative power. Thiacloprid achieves an AUC score of 0.95, 0.93 by Imidacloprid, 0.90 by Thiamethoxam; Nitenpyram achieves score of 0.91, 0.94 by Tetrahydrofolate, and 0.92 by Dihydrofolate. This critical analysis validates that the biosensor is effectively distinguished between other related molecular signatures which is considered as a key requirement in real-world biosensing.

**Fig. 12:**
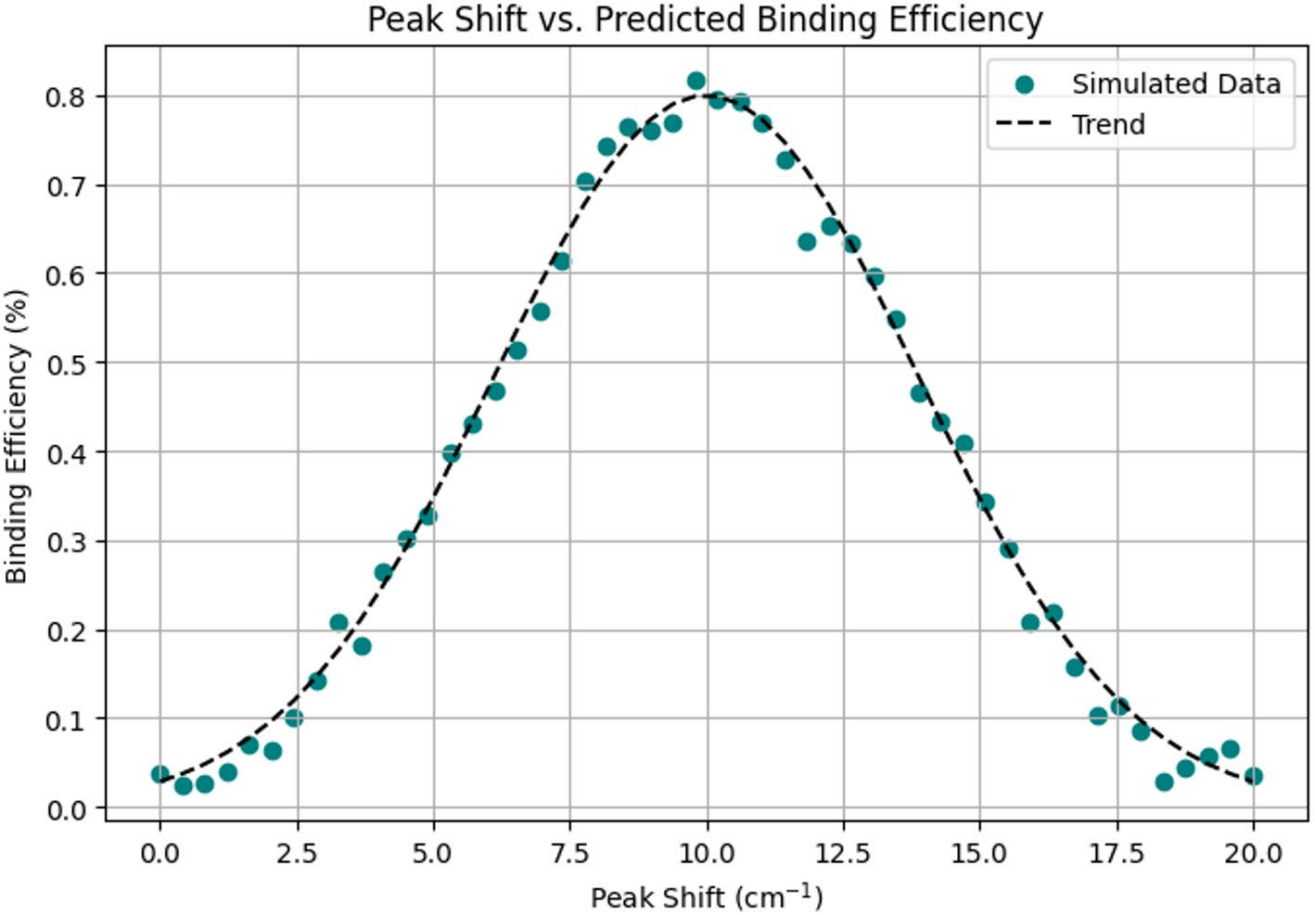
Peak shift vs. predicted binding efficiency

Figure 12 establishes the strong relationship between spectral peak shifts and the predicted molecular binding efficiency. As the peak shift increases from 0 to 10 cm^− 1^, there is a notable rise in binding efficiency, showcasing the biosensor’s heightened sensitivity to molecular interactions at different shifts. This clear trend validates the model’s capacity to capture meaningful physical changes in the sensor’s plasmonic environment.

**Fig. 13:**
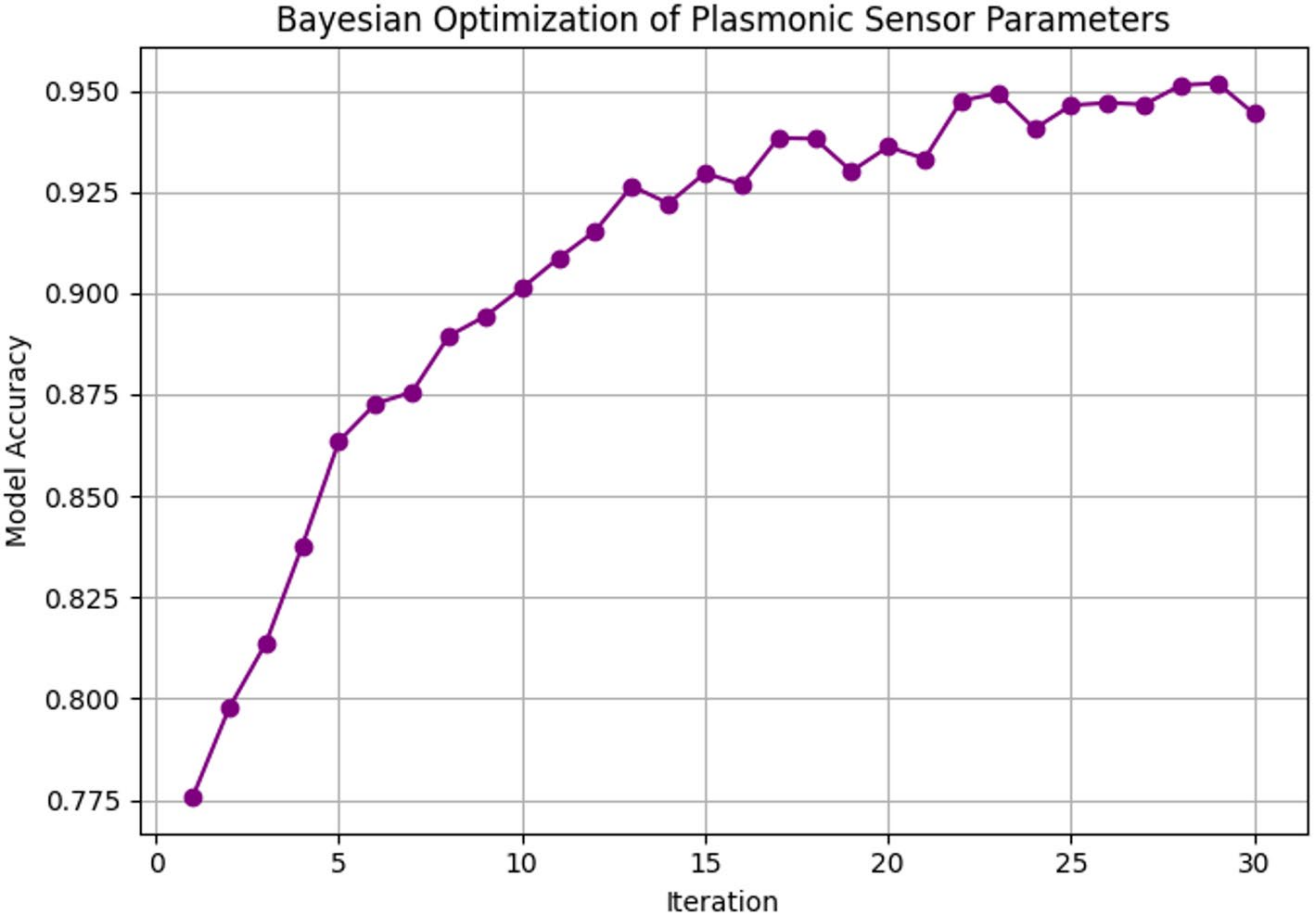
Bayesian optimization of plasmonic sensor parameters

The performance of the Bayesian optimization algorithm over biosensor parameters is illustrated in Fig. 13. The accuracy of the model gradually starts at 75% and steadily improves over successive iterations, reaching a stable value near 92% at around 20 iterations. This trend indicates that the optimization algorithm identifies near-optimal sensor configurations that maximize performance. This trajectory emphasizes the efficacy of ML-guided optimization in achieving high-performance biosensor configurations more efficiently than traditional trial-and-error methods.

### Comparative analysis

**Fig. 14:**
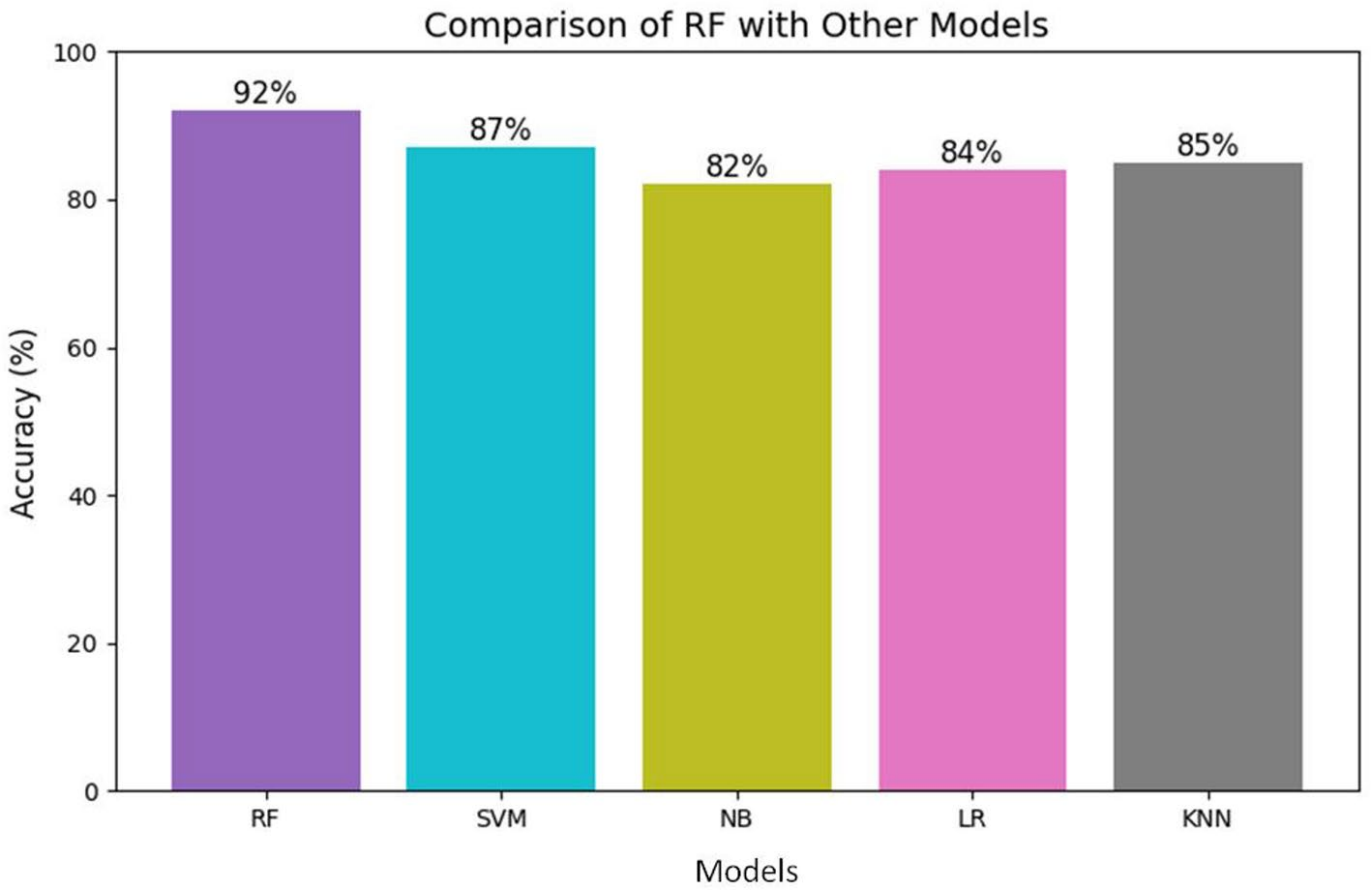
Comparison of RF with other classifier models

The performance of RF is compared with other classifier models like Support Vector Machine (SVM), Naïve Bayes (NB), Linear Regression (LR), and K-Nearest Neighbor (KNN) in terms of plasmonic or biosensing behavior, and the results are graphically represented in Fig. 14. The plot shows how the random forest model has outperformed the other baseline options through the acquisition of 92% accuracy. SVM follows RF with 87% while NB lags at 82%. These results underline the robustness and reliability of RF in classifying complex feature spaces, which is typical in biosensor signal data.

**Fig. 15:**
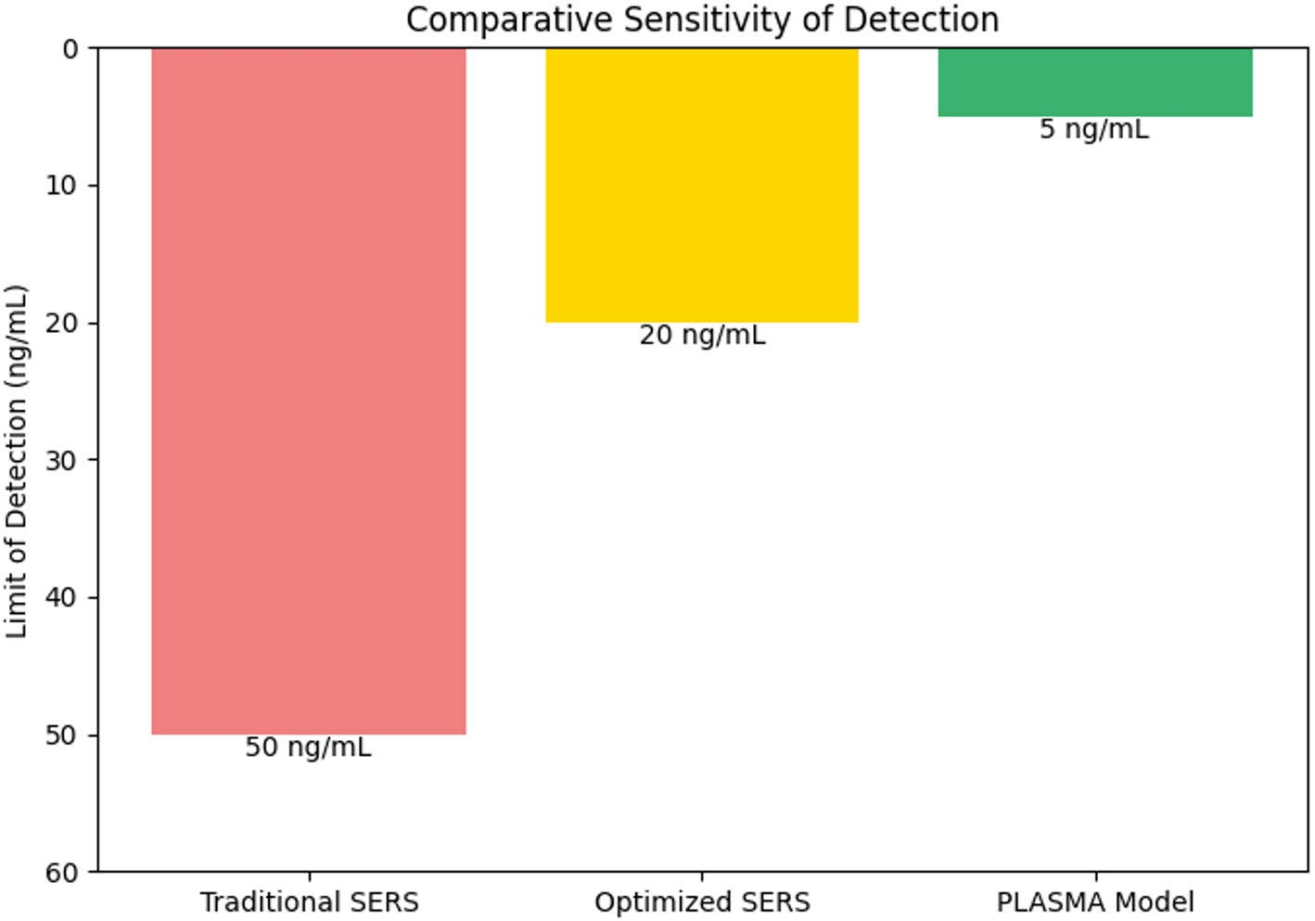
Comparative analysis of sensitivity between different models

**Fig. 16:**
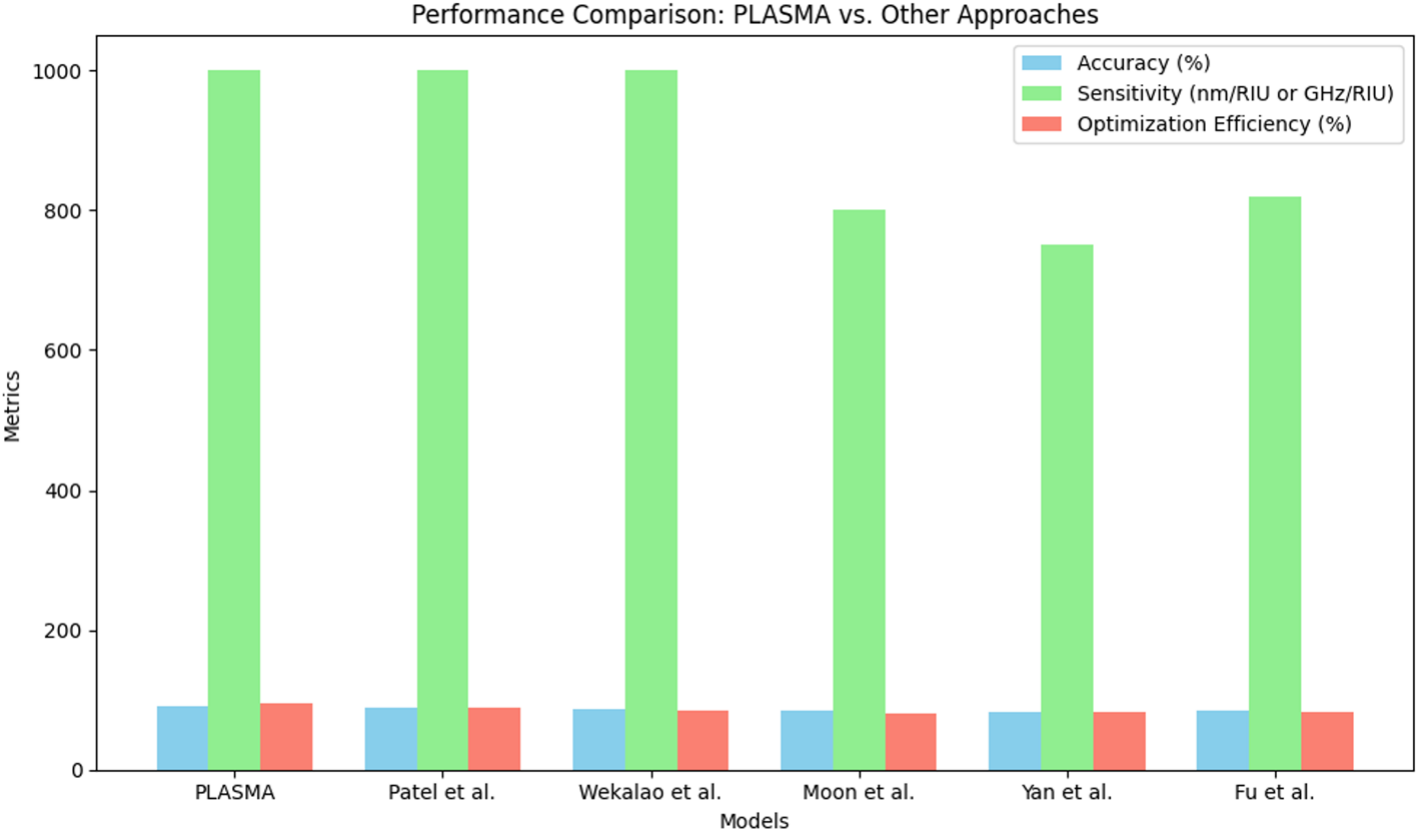
Comparative analysis of SERA with other plasmonic biosensor optimization models

The comparative analysis in Fig. 15 highlights the improvement in detection sensitivity across different biosensor configurations. The traditional SERS-based sensor achieves a limit of detection (LOD) of approximately 50 ng/mL, while optimized SERS scores a LOD of approximately 20 ng/mL. Notably, the SERA technique pushes the sensitivity as low as 5 ng/mL, demonstrating the transformative impact of ML-driven optimization, which in turn improves the ultra-sensitive detection, surpassing the conventional methods. This validation proves the superiority of the trace-level molecular detection of the SERA technique in biosensing applications. Finally, in order to prove the superiority of the SERA over other optimization models discussed in the literature section, a comparative analysis is made on factors like accuracy, sensitivity, and optimization efficiency, and the results are given in Table 5; Fig. 16. The plot illustrates that the proposed model outperforms the other biosensor optimization approaches across all the metrics. With an accuracy of 92%, it slightly exceeds the best-performing baseline model proposed by Patel et al. [20]. The model showcased superior optimization efficiency of 95% and sensitivity parity of 1000 nm/RIU, indicating top-tier detection and a robust tool for real-world biosensor applications.

**Table 5:** Comparison of SERA with other plasmonic biosensor optimization models

Model	Accuracy (%)	Sensitivity (nm/RIU)	Optimization efficiency (%)
SERA (Proposed)	92	1000 nm/RIU	95
Patel et al., [20]	89	1000 nm/RIU	88
Wekalao et al., [22]	87	1000 GHz/RIU	85
Moon et al., [16]	85	800 nm/RIU	80
Yan et al., [23]	82	750 nm/RIU	82
Fu et al., [25]	84	820 nm/RIU	83

## Conclusion

At present, SPR and SERS have gained immense attention in various fields like medical diagnosis, environmental monitoring, and bio photonics. While the conventional techniques rely on empirical optimization, which is time-consuming, the evolution of AI in the field of plasmonic biosensors has opened access to various research works. One such machine learning-driven optimization technique called SERA is proposed in this paper for ultra-sensitive detection in plasmonic biosensor. The data and simulation from the SERS-DB database are first processed in the pre-processing stage through techniques like baseline correction, noise reduction, and normalization. The processed data is then extracted for useful features like intensity, spatial details, and peak shifts using PCA and the Local Maxima search approach. RF, being the optimal ML classifier option, is chosen for the SERA model for spectral and pattern recognition. The sensor parameters are then optimized using Bayesian optimization, followed by validation and deployment. With a total sample of 420 for training and 180 for the testing phase, 6 classes like Thiacloprid, Imidacloprid, Thiamethoxam, Nitenpyram, Tetrahydrofolate, and Dihydrofolate, belonging to molecular types like pesticides and biological molecules, were taken for the primary evaluation purpose.

The model showcased good performance in accuracy and loss metric during both the phases and upon interpretation of the model on resonance shift, intensity, and binding efficiency across 6 classes, Tetrahydrofolate achieved the highest resonance shift of 35 cm^− 1^ and intensity of 75 a.u. highlighting stronger molecular interactions and revealing an overall error tolerance of approximately 5%. This low deviation confirms robustness and reliability of developed framework. In the standard metrics, an accuracy of 92%, precision & recall of 90%, and F1-score of 92% were obtained. In the ROC analysis, the SERA model excelled with an overall score of around 0.90 in all 6 classes. Upon comparison of the random forest classifier with other models like NB, SVM, and LR, the classifier attained a high accuracy of 92%, making it feasible for complex feature spaces. While this present concept establishes a solid foundation for AI-driven plasmonic biosensor optimization, future research focuses on expanding the dataset to include more diverse biomolecular classes and real-time experimental validations. Also, apart from six molecules considered, the model can be expanded to a wide range of analytes, pesticides, biological, and pharmaceutical marker classes (neonicotinoids- chlorpyrifos, malathion; antibiotics-tetracycline, ciprofloxacin; protein biomarkers – CRP, PSA; and biomarkers – dopamine, glucose). Further, the inclusion of DL architectures like CNN can further improve the feature extraction from complex spectral data. Additionally, integrating the SERA model with microfluidic systems and wearable biosensors can open avenues for real-world, point-of-care diagnostics. Finally, integrating Bayesian with reinforcement learning further refines the sensor parameters tuning process of dynamic and evolving sensing environments.

## Data Availability

The datasets used and/or analysed during the current study available from the corresponding author on reasonable request.

## Code availability

The relevant code with the manuscript is also available and would be available, if will be asked to do so later.

## Declarations

### Clinical trial

If your study is a clinical trial, then please provide the necessary registration details (registry, trial registration number, and data of registration): Not Applicable.
